# The Metric System in Medicine

**Published:** 1880-02

**Authors:** 


THE METRIC SYSTEM IN MEDICINE.—We have not taken any measures to ascertain how far the druggists of this city or in the country have provided themselves with the means for filling prescriptions written in accordance with the metric system of weights and measures. We are quite sure, however, that in this city, and probably in all the more important towns in the country, they are so far prepared that no danger would attend the full, practical adoption of the system by physicians in their daily routine of practice. That the system has many advantages over the one hitherto in use, is manifest to all who have given it a fair trial. First. It enables us to express all the ingredients of a prescription in the same measure, instead of mixing grains, scruples, drachms and ounces. Second. It enables us to make a much more minute and accurate proportion of ingredients in prescriptions containing one or more articles, the doses of which are but the small fraction of a grain. For instance, in prescribing the bi-chloride of mercury with the tincture of cinchona: to get a proper relative dose of each for ordinary tonic and alterant purposes, there should be only one grain of the bi-chloride to four ounces of the tincture. But to order a four-ounce vial of medicine for a child of such age that the proper dose would be only eight or ten minims three times a day, looks like making a prescription that is to last something less than a year; yet we have always felt unsafe to order less than one grain of anything under the old system of weights. But with weights and apparatus for weighing grams, decigrams, centigrams, and milligrams, the various ingredients in a prescription may be adjusted with the utmost minuteness and accuracy. What we have said of the grain, applies equally to the scruple, drachm and ounce. We have a well understood sign in the "ss" for half a drachm or half an ounce, but none for one-third or one-quarter of a drachm or ounce. Therefore, while we can readily write ℥iiss, if we desire to express another ingredient in the same prescription at two ounces and a quarter, we must write it ʒxviii; if another in two and one-third ounces, we should be obliged to express it in ℈LVi. Third. The use of the decimal in the multiplication and division of quantities in the metric system is very much more simple and convenient than any other, as soon as we become accustomed to its use. It was not our purpose, however, at the present time, to discuss the advantages or disadvantages of the metric system, but rather to urge the importance of having the profession decide with more promptness whether to adopt the system or not. It has been sanctioned and its adoption recommended by most of the important medical organizations, from the American Medical Association to many of the local societies. But at the present time its practical adoption is in a very mixed condition. Some medical teachers use it, and others, even in the same faculty, do not. Some writers and publishers adhere to the old system; others adopt the new; and still others give both. And the same is true of the individual practitioners.

This uncertain and mixed state of things should not continue long. It is already beginning to discourage some who had from the first earnestly adopted the metric system, under the expectation that such adoption was speedily to become general. If it is really desirable to introduce this system into universal use in our profession, steps should be taken without delay to secure some concert of action on the part of the teachers in medical colleges, the editors of medical periodicals, and the writers and publishers of medical books. The parties composing these three classes have it in their power to completely control the whole matter. If those engaged in medical teaching would use the language of the metric system in giving instruction; those editing medical journals, for the first year give all formulas in both systems, and after that period only in the metric; and those writing and publishing new works, do the same; in less than five years the new system would become as familiar to the great mass of the profession as the old one is now, and the transition would be practically complete.

Could not such concert of action as we suggest be easily obtained? There is a National Association of American Medical Editors, and an American Medical College Association, both of which are expected to hold meetings in close connection with the annual meeting of the American Medical Association, in New York, the first week in June next; and we suggest that this subject would constitute an important topic for full consideration at those meetings. The American Association of Medical Editors was organized in 1869, and has held a short meeting annually since, doing little else than to listen to an address from the president, and elect new officers. We suggest to all our brother editors the propriety of giving this subject some attention, and coming together at the next meeting of our association, prepared to take definite and controlling action concerning it. We say *controlling* action, because the unanimous adoption of the system by the medical periodical press would soon induce co-operative action on the part of both medical teachers and book publishers.

